# Automated Detection of Infectious Disease Outbreaks in Hospitals: A Retrospective Cohort Study

**DOI:** 10.1371/journal.pmed.1000238

**Published:** 2010-02-23

**Authors:** Susan S. Huang, Deborah S. Yokoe, John Stelling, Hilary Placzek, Martin Kulldorff, Ken Kleinman, Thomas F. O'Brien, Michael S. Calderwood, Johanna Vostok, Julie Dunn, Richard Platt

**Affiliations:** 1 Division of Infectious Diseases and Health Policy Research Institute, University of California Irvine School of Medicine, Irvine, California, United States of America; 2 Channing Laboratory, Brigham and Women's Hospital and Harvard Medical School, Boston, Massachusetts, United States of America; 3 Department of Medicine, Brigham and Women's Hospital, Boston, Massachusetts, United States of America; 4 Department of Clinical and Population Health Research, University of Massachusetts Medical School - Worcester, Worcester, Massachusetts, United States of America; 5 Department of Population Medicine, Harvard Medical School and Harvard Pilgrim Health Care Institute, Boston, Massachusetts, United States of America; 6 Department of Epidemiology, Rollins School of Public Health, Atlanta, Georgia, United States of America; Free University of Brussels, Belgium

## Abstract

**Background:**

Detection of outbreaks of hospital-acquired infections is often based on simple rules, such as the occurrence of three new cases of a single pathogen in two weeks on the same ward. These rules typically focus on only a few pathogens, and they do not account for the pathogens' underlying prevalence, the normal random variation in rates, and clusters that may occur beyond a single ward, such as those associated with specialty services. Ideally, outbreak detection programs should evaluate many pathogens, using a wide array of data sources.

**Methods and Findings:**

We applied a space-time permutation scan statistic to microbiology data from patients admitted to a 750-bed academic medical center in 2002–2006, using WHONET-SaTScan laboratory information software from the World Health Organization (WHO) Collaborating Centre for Surveillance of Antimicrobial Resistance. We evaluated patients' first isolates for each potential pathogenic species. In order to evaluate hospital-associated infections, only pathogens first isolated >2 d after admission were included. Clusters were sought daily across the entire hospital, as well as in hospital wards, specialty services, and using similar antimicrobial susceptibility profiles. We assessed clusters that had a likelihood of occurring by chance less than once per year. For methicillin-resistant *Staphylococcus aureus* (MRSA) or vancomycin-resistant enterococci (VRE), WHONET-SaTScan–generated clusters were compared to those previously identified by the Infection Control program, which were based on a rule-based criterion of three occurrences in two weeks in the same ward. Two hospital epidemiologists independently classified each cluster's importance. From 2002 to 2006, WHONET-SaTScan found 59 clusters involving 2–27 patients (median 4). Clusters were identified by antimicrobial resistance profile (41%), wards (29%), service (13%), and hospital-wide assessments (17%). WHONET-SaTScan rapidly detected the two previously known gram-negative pathogen clusters. Compared to rule-based thresholds, WHONET-SaTScan considered only one of 73 previously designated MRSA clusters and 0 of 87 VRE clusters as episodes statistically unlikely to have occurred by chance. WHONET-SaTScan identified six MRSA and four VRE clusters that were previously unknown. Epidemiologists considered more than 95% of the 59 detected clusters to merit consideration, with 27% warranting active investigation or intervention.

**Conclusions:**

Automated statistical software identified hospital clusters that had escaped routine detection. It also classified many previously identified clusters as events likely to occur because of normal random fluctuations. This automated method has the potential to provide valuable real-time guidance both by identifying otherwise unrecognized outbreaks and by preventing the unnecessary implementation of resource-intensive infection control measures that interfere with regular patient care.

*Please see later in the article for the Editors' Summary*

## Abstract 2

Susan Huang and colleagues describe an automated statistical software, WHONET-SaTScan, its application in a hospital, and the potential it has to identify hospital infection clusters that had escaped routine detection.

## Editors' Summary

**Background:**

Admission to a hospital is often a life-saving necessity—individuals injured in a road accident, for example, may need immediate medical and surgical attention if they are to survive. Unfortunately, many patients acquire infections, some of which are life-threatening, during their stay in a hospital. The World Health Organization has estimated that, globally, 8.7% of hospital patients develop hospital-acquired infections (infections that are identified more than two days after admission to hospital). In the US alone, 2 million people develop a hospital-acquired infection every year, often an infection of a surgical wound, or a urinary tract or lung infection. Infections are common among hospital patients because increasing age or underlying illnesses can reduce immunity to infection and because many medical and surgical procedures bypass the body's natural protective barriers. In addition, poor infection control practices can facilitate the transmission of bacteria—including meticillin-resistant *Staphylococcus aureus* (MRSA) and vancomycin-resistant enterococci (VRE)—and other infectious agents (pathogens) between patients.

**Why Was This Study Done?:**

Sometimes, the number of cases of hospital-acquired infections increases unexpectedly or a new infection emerges. Such clusters account for relatively few health care–associated infections, but, because they may arise from the transmission of a pathogen within a hospital, they need to be rapidly identified and measures implemented (for example, isolation of affected patients) to stop transmission if an outbreak is confirmed. Currently, the detection of clusters of hospital-acquired infections is based on simple rules, such as the occurrence of three new cases of a single pathogen in two weeks on the same ward. This rule-based approach relies on the human eye to detect infection clusters within microbiology data (information collected on the pathogens isolated from patients), it focuses on a few pathogens, and it does not consider the random variation in infection rates or the possibility that clusters might be associated with shared facilities rather than with individual wards. In this study, the researchers test whether an automated statistical system can detect outbreaks of hospital-acquired infections quickly and accurately.

**What Did the Researchers Do and Find?:**

The researchers combined two software packages used to track diseases in populations to create the WHONET-SaTScan cluster detection tool. They then compared the clusters of hospital-acquired infection identified by the new tool in microbiology data from a 750-bed US academic medical center with those generated by the hospital's infection control program, which was largely based on the simple rule described above. WHONET-SaTScan found 59 clusters of infection that occurred between 2002 and 2006, about three-quarters of which were identified by characteristics other than a ward-based location. Nearly half the cluster alerts were generated on the basis of shared antibiotic susceptibility patterns. Although WHONET-SaTScan identified all the clusters previously identified by the hospital's infection control program, it classified most of these clusters as likely to be the result of normal random variations in infection rates rather than the result of “true” outbreaks. By contrast, the hospital's infection control department only identified three of the 59 statistically significant clusters identified by WHONET-SaTScan. Furthermore, the new tool identified six previously unknown MRSA outbreaks and four previously unknown VRE outbreaks. Finally, two hospital epidemiologists (scientists who study diseases in populations) classified 95% of the clusters detected by WHONET-SaTScan as worthy of consideration by the hospital infection control team and a quarter of the clusters as warranting active investigation or intervention.

**What Do These Findings Mean?:**

These findings suggest that automated statistical software should be able to detect clusters of hospital-acquired infections that would escape detection using routine rule-based systems. Importantly, they also suggest that an automated system would be able to discount a large number of supposed outbreaks identified by rule-based systems. These findings need to be confirmed in other settings and in prospective studies in which the outcomes of clusters detected with WHONET-SaTScan are carefully analyzed. For now, however, these findings suggest that automated statistical tools could provide hospital infection control experts with valuable real-time guidance by identifying outbreaks that would be missed by routine detection methods and by preventing the implementation of intensive and costly infection control measures in situations where they are unnecessary.

**Additional Information:**

Please access these Web sites via the online version of this summary at http://dx.doi.org/10.1371/journal.pmed.1000238.

- The World Health Organization's Prevention of Hospital-Acquired Infections, A Practical Guide contains detailed information on all aspects of hospital-acquired infections
- MedlinePlus provides links to information on infection control in hospitals (in English and Spanish)
- The US Centers for Disease Control and Prevention also provides information on infectious diseases in health care settings (in English and Spanish)
- The WHONET/Baclink software and the SatScan software, the two components of WHONET-SaTScan are both available on the internet (the WHONET-SaTScan cluster detection tool is freely available as part of the version of WHONET/BacLink released June 2009)

## Introduction

Although hospital-associated outbreaks of infection account for a small proportion of health care–associated infections [1]–[4], the fact that they typically result from transmission within health care facilities means that timely identification is essential for investigation and effective response. Current detection methods rely heavily on temporal or spatial clustering of specific pathogens. Such monitoring usually involves case counting and subjective judgment to adjudicate whether a cluster is occurring. For multidrug-resistant organisms (MDROs), such as methicillin-resistant *Staphylococcus aureus* (MRSA), rule-based criteria (e.g., three cases within 2 wk in the same ward) are often used to define a cluster. For example, Mellmann et al. used a definition of two cases in 2 wk with identical spa types to define a MRSA outbreak [5].

Ad hoc and rule-based criteria are subject to error—both in defining random variation as a cluster and in failing to identify clusters owing to hospital transmission that do not meet specified rules. Reliance on the human eye to filter daily microbiology data and detect clusters among hundreds of pathogens can lead to a high failure rate. In addition, reliance on subjective judgment by infection control professionals for cluster detection can lead to interhospital variability and incorrect identification. Because clusters (perceived or real) engender intensive investigation and possible intervention, identification of false clusters can waste valuable resources and dilute attention to real problems.

Microbiology-based cluster detection systems should use automated statistical methods to optimize cluster identification, lessen surveillance burden, and expand cluster detection to all pathogens across all hospital locations and services. It should automatically assess whether pathogens in a cluster had similar antimicrobial susceptibility patterns that would suggest clonality and a common source. Requirements for a useful system include (1) automatic and timely generation of alerts of clusters, (2) sufficient sensitivity to detect clinically significant clusters identified through routine surveillance methods, and (3) sufficient positive predictive value to avoid an excessive number of false alerts that could generate unnecessary investigation and intervention.

## Methods

### Ethics Statement

This study was approved by the Brigham & Women's Hospital (BWH) Institutional Review Board.

### Study Population and Datasets

BWH is a 750-bed academic medical center. It provides neonatal and adult medical care with intensive care and oncology patient populations. Its electronic data repository contains finalized microbiology data from 1987 to present. The microbiology data repository includes patient identifiers, ward, and clinical service at the time of specimen collection, collection date and specimen source, and hospital admission date. Antimicrobial susceptibility testing is based on Clinical and Laboratory Standards Institute (CLSI) standards [6].

The entire microbiology data repository was used to identify the first positive result per patient for a specific bacterial or fungal species since 1987. The dataset was further restricted to isolates representing hospital-associated acquisition (All Organism Nosocomial Dataset) by limiting isolates to those obtained >2 d after hospital admission. In addition, a second dataset was created that limited pathogens to organism species associated with hospital transmission on the basis of published literature (Priority Pathogen Nosocomial Dataset) (Table 1). Because of national surveillance related to multidrug-resistant bacteria, we additionally assessed MRSA and VRE.

**Table 1:** Priority pathogens previously described in hospital-associated clusters.

Pathogen
*Acinetobacter* sp.
*Alcaligenes* sp.
*Aspergillus* sp.
*Bacteroides* sp.
*Burkholderia* sp.
*Candida* sp.
*Chromobacterium* sp.
*Chryseobacterium* sp.
*Citrobacter* sp..
*Enterobacter* sp.
*Enterococcus* sp.
Each species regardless of resistance profile
VRE
*Escherichia* sp.
*Fusarium* sp.
Group A *Streptococcus*
*Haemophilus* sp.
*Klebsiella* sp.
*Legionella* sp.
*Malassezia* sp..
*Mycobacterium* sp.
*Oligella* sp.
*Pantoea* sp.
*Proteus* sp.
*Pseudomonas* sp.
*Rhizopus* sp.
*Salmonella* sp.
*Serratia* sp.
*S. aureus*
All isolates regardless of resistance profile
MRSA
*Stenotrophomonas* sp.
*Torulopsis* sp.

All species individually assessed within genus.

### Automated Cluster Detection Tool

We integrated two freely available software packages used for public health epidemiology. WHONET/BacLink software is available from the World Health Organization (WHO) Collaborating Centre for Surveillance of Antimicrobial Resistance for management and descriptive analysis of microbiology data [7]. BacLink is a data-conversion utility that standardizes data from existing microbiology systems into WHONET formats. WHONET/BacLink is used by >1,000 laboratories world-wide. SaTScan was originally developed for geographical disease surveillance to assess the statistical significance of community cancer clusters [8]–[10]. The software was subsequently enhanced and applied to early detection of infectious disease outbreaks [11],[12]. We integrated the space-time permutation scan statistic in SaTScan into the WHONET analysis module to create the WHONET-SaTScan cluster detection tool, which is now freely available as part of WHONET/BacLink as of June 2009 [7].

For hospital surveillance, “spatial” locations consisted of individual wards and services (e.g., medicine, oncology). In addition, we evaluated groups of wards or services sharing in patient care (e.g., cardiology and cardiac surgery services), regardless of physical proximity. Antimicrobial resistance profile was also used as a spatial location to detect clusters of specific pathogens that had identical patterns of nonsusceptibility to routinely tested antibiotics. Using only case data, the space-time permutation scan statistic looks for space-time interaction clusters, adjusting for purely temporal and purely spatial variation [12]. The space-time cluster with the maximum likelihood is the cluster least likely due to chance. For each pathogen in the Priority Pathogen Nosocomial Dataset, a separate set of analyses were done for wards, services, and antimicrobial resistance pattern. It is important to note that this method will be subject to human-influenced variation, such that if one ward expanded in volume because of increasing bed size, then this increase may trigger a cluster alert in the ward-based analysis.

Surveillance for hospital-wide clusters was performed by replacing “space” in the space-time permutation scan statistic with “pathogen.” This assessment was applied to the All Organism Nosocomial Dataset, to detect clusters that were not explained by a general simultaneous increase in all pathogens, as might occur with new diagnostics that enhance overall pathogen detection by culture systems or increased culturing because of changes in physician practice. Similarly, the WHONET-SaTScan tool adjusts for (i.e., would not detect) weekly or seasonal increases that occurred simultaneously across all “spatial” locations, such as all wards in the ward-based analyses, or all nosocomial pathogens in the hospital-wide analyses. However, nosocomial increases in specific wards would be detected in the ward-based analyses and increases in specific pathogens would be detected in the hospital-wide analyses.

Each pathogen-specific set of analyses was performed “daily” from 2002 to 2006, mimicking real-time prospective surveillance among all patients admitted to BWH during this time period. Within each set, the method adjusts for multiple testing inherent in the many combinations of wards, services, pathogens, and resistance patterns considered, and for the large number of days evaluated.

### Selecting WHONET-SaTScan Parameters

Datasets from 2001 were used to select software parameters. The maximum number of days over which isolates could contribute to the initial determination that a cluster had occurred was set to 60 d. This parameter setting was based principally upon biologic plausibility of ongoing transmission due to a common source, as well as the practical ability to respond and intervene. For example, if a cluster alert is signaled in December based upon two cultures—one in the preceding January and one in December—one might conclude that notification was unhelpful since the prolonged time lapse since the January event makes it unlikely that current investigation or intervention would be meaningful. A maximum span of 60 d was chosen after the 2001 assessment of 30, 60, and 90 d revealed increased cluster detection with 60 d, but minimal improvement with 90 d.

Because an ongoing cluster can span many months, we did not restrict the time that a cluster could persist (continue to generate alerts). If new cases continued to occur, they would generate alerts as long as the statistical threshold was met. For presentation purposes, alerts from the same cluster were combined into a summary report that included the number of observed versus expected cases across the duration of the cluster, the time from the first culture of the cluster until the first alert, and the total duration of the cluster. Thus, a cluster could be represented by a single alert or a set of overlapping alerts that would signal a potential outbreak. WHONET-SaTScan scanned for clusters on a daily basis by comparing the number of cases in a specific time window to the expected number based on the 365 d prior to the day of analysis. We selected statistical thresholds for detecting clusters on the basis of recurrence intervals [13]. The recurrence interval is the expected frequency of falsely identifying a cluster by chance alone. A recurrence interval of 100 d means that a cluster as unusual as the identified cluster would occur by chance approximately once every 100 d. We evaluated recurrence intervals of 200, 365, and 1,000 d using the 2001 test dataset and compared the results to 2001 clusters previously identified by routine infection control surveillance and confirmed by genetic typing of isolates. A recurrence interval threshold of ≥365 d was selected, because recurrence intervals <365 d were not associated with known clusters and were of limited epidemiologic significance based on available microbiology data and medical records.

### WHONET-SaTScan Assessment Dataset

Through simulation, we mimicked daily prospective cluster detection from 2002 to 2006 by adding each day's experience and repeating the analyses, as would occur in real time. Once an alert was generated, alerts for the same cluster were generated on subsequent days only if cases increased and the statistical threshold was still met. Alert reports included the organism, alert type (e.g., ward, service, antibiotic profile, or hospital-wide), date of the first alert for that cluster, date of the first specimen of that cluster, observed and expected number of cases, and statistical significance (i.e., the recurrence interval). Line-item culture results, including date, location, and patient identifiers, were also generated for each cluster alert.

### Comparing WHONET-SaTScan to Routine Infection Control Methods

Detailed infection control records from 2002 to 2006 were reviewed for clusters on the basis of routine surveillance. We compared WHONET-SaTScan results to two types of identified clusters: (1) those with a known epidemiologic source and identical strains by genetic typing, and (2) clusters of MRSA and VRE defined by rule-based criteria involving ≥3 nosocomial cases in a ward within 2 wk. These rule-based clusters triggered ward-wide precautions involving alerts sent to nursing and physician leadership, admission and weekly screening of all ward patients (nares cultures for MRSA and rectal cultures for VRE), and use of gloves for all patient contact until no new cases were identified for a 4-wk period or until all cases were discharged from the ward. We compared the “three in 2-wk” criteria for MRSA and VRE clusters to statistically significant clusters identified by WHONET-SaTScan.

### Assessing Usefulness of Cluster Alerts

We assessed the usefulness and interpretability of the alert notification system by creating daily alert reports using the 2002–2006 dataset and providing these to two hospital epidemiologists who independently reviewed each day's report in sequence and indicated their level of concern and recommended actions on a survey form. The survey asked whether the alert was considered worth knowing about and which of four types of responses (ignore, watch and wait, investigate with detailed chart review, and actively intervene with ward-wide cluster precautions) was recommended. Characteristics of clusters (size, type, recurrence interval) associated with active intervention were assessed using Fisher exact tests.

Surveys were completed with the knowledge of medical record details (chronological ward and room assignments, service, culture source, and antimicrobial susceptibility profile) that would have been available in real time from cases. The concordance of survey responses from the two hospital epidemiologists for initiating either an investigation or active intervention was assessed by a kappa statistic. Survey responses were also combined into a summary description that used the more intensive response recommended by either hospital epidemiologist.

## Results

The All Organism Nosocomial Dataset from 2002 to 2006 included 298 organism codes and 32,482 isolates. The Priority Pathogen Nosocomial Dataset included 41% of those isolates. All but one cluster involved priority pathogens (Table 1).

Summary characteristics of WHONET-SaTScan clusters are found in Table 2. A total of 59 clusters were identified in the 5-y dataset, giving an average of 12 clusters per year. The mean and median cluster sizes were 6 and 4, respectively. Detailed descriptions of each cluster are found in Table 3. Two clusters were identified by two different spatio-temporal analyses (e.g., ward-level and service-level).

**Table 2:** Characteristics of detected clusters, 2002–2006.

Cluster Characteristics	*n* (%)
**Total clusters**	59
**Annual clusters: median (range)**	12 (7–16)
**Year**	
2002	14 (23.7)
2003	7 (11.9)
2004	10 (16.9)
2005	12 (20.3)
2006	16 (27.1)
**Organisms**	
Gram positive	21 (35.6)
Gram negative	31 (52.5)
Fungi	7 (11.9)
**Alert type** a	
Hospital-wide	11 (18.0)
Ward(s)	16 (26.2)
Service(s)	8 (13.1)
Antibiotic profile	26 (42.6)
**Size (** ***n*** ** cases)**	
1–2	12 (20.3)
3–5	27 (45.8)
6–10	11 (18.6)
>10	9 (15.3)

a Two clusters were identified by two different types of alerts.

**Table 3:** Potential hospital-associated clusters detected using WHONET-SaTScan automated system, 2002–2006.

Organism	Signal Type	Observed Cases	Expected Cases	Days to First Signal^a^	Span of Signals^b^	Cluster Year	Recurrence Interval^c^	Previously Identified by Infection Control
**Gram-positive bacteria**								
*E. faecalis*	Antibiotic profile	4	0.6	18	25	2004	667	N
*E. faecalis*	Service	4	0.6	10	17	2005	1,429	N
*E. faecium*	Antibiotic profile	3	0.3	1	20	2006	1,429	N
*E. faecium* (VRE)	Antibiotic profile	5	1.0	13	57	2002	625	N
*E. faecium* (VRE)	Antibiotic profile	6	1.3	31	29	2002	769	N
*E. faecium* (VRE)	Antibiotic profile	4	0.6	42	18	2003	1,429	N
*E. faecium* (VRE)	Antibiotic profile	2	0.14	29	17	2004	500	N
*Propionibacterium acnes*	Hospital-wide	10	2.7	11	7	2006	1,429	N
*S. aureus*	Antibiotic profile	2	0.0	0	5	2002	2,000	N
*S. aureus*	Ward	3	0.1	0	2	2003	833	N
*S. aureus*	Ward	3	0.1	1	1	2003	833	N
*S. aureus*	Ward	7	1.1	6	16	2004	667	N
*S. aureus*	Antibiotic profile	4	0.3	2	4	2006	385	N
*S. aureus* (MRSA)	Antibiotic profile	14	2.8	1	67	2002	10,000	N
*S. aureus* (MRSA)	Ward	3	0.1	0	6	2005	5,000	N
*S. aureus* (MRSA)	Ward	8	1.4	6	54	2004	10,000	Y
*S. aureus* (MRSA)^d^	Ward	6	0.91	33	15	2005	833	N
*S. aureus* (MRSA)^d^	Service	4	0.44	8	5	2005	625	N
*S. aureus* (MRSA)	Antibiotic profile	2	0.04	6	4	2005	667	N
*S. aureus* (MRSA)	Service	6	1.05	8	9	2006	2,500	N
*S. aureus* (MRSA)	Antibiotic profile	2	0.09	4	3	2006	435	N
*Streptococcus*, Group A	Hospital-wide	3	0.2	0	15	2005	3,333	N
**Gram-negative bacteria**								
*A. baumannii*	Multi Service	4	0.8	2	24	2002	5,000	N
*A. baumannii*	Hospital-wide	5	0.5	1	6	2002	588	N
*A. baumannii* e	Antibiotic profile	15	7.5	18	52	2004	10,000	Y
*A. baumannii* e	Hospital-wide	20	8.3	3	57	2004	625	Y
*A. baumannii*	Ward	4	0.6	3	9	2006	2,000	N
*Bacteroides fragilis*	Service	2	0.2	4	1	2006	500	N
*B. cepacia*	Hospital-wide	15	3.8	6	60	2005	10,000	Y
*C. freundii*	Antibiotic profile	2	0.1	4	27	2006	10,000	N
*E. aerogenes*	Antibiotic profile	3	1.8	2	26	2006	909	N
*E. cloacae*	Antibiotic profile	3	0.0	1	28	2002	10,000	N
*E. cloacae*	Hospital-wide	11	2.7	2	6	2002	1,250	N
*E. cloacae*	Antibiotic profile	4	0.5	4	2	2005	476	N
*E. cloacae*	Service	11	3.6	14	46	2005	370	N
*E. cloacae*	Antibiotic profile	4	0.3	6	33	2006	769	N
*E. cloacae*	Multiward	5	0.8	20	36	2006	2500	N
*E. cloacae*	Antibiotic profile	27	4.3	42	163	2006	10,000	N
*E. coli*	Antibiotic profile	4	0.5	3	34	2002	476	N
*E. coli*	Antibiotic profile	6	1.1	6	9	2005	2,500	N
*H. influenzae*	Hospital-wide	13	4.2	18	14	2004	455	N
*H. influenzae*	Antibiotic profile	6	1.0	8	52	2006	5,000	N
*K. oxytoca*	Antibiotic profile	2	0.2	24	12	2004	1111	N
*K. oxytoca*	Antibiotic profile	2	0.2	0	30	2006	10,000	N
*K. pneumoniae*	Ward	3	0.2	3	16	2003	909	N
*P. (Entero.) agglomerans*	Hospital-wide	4	0.2	4	2	2002	400	N
*P. aeruginosa*	Multi Service	5	0.6	4	7	2002	833	N
*P. aeruginosa*	Antibiotic profile	3	0.2	2	7	2004	476	N
*P. aeruginosa*	Ward	2	0.0	1	3	2005	476	N
*S. marcescens*	Antibiotic profile	3	0.4	34	10	2002	435	N
*S. marcescens*	Multi Service	4	0.5	12	4	2003	556	N
*S. marcescens*	Hospital-wide	10	2.8	10	3	2004	2,500	N
*S. marcescens*	Antibiotic profile	11	1.4	21	118	2006	10,000	N
*S. maltophilia*	Ward	3	0.3	6	9	2006	2,000	N
**Fungi**								
*A. fumigatus*	Hospital-wide	7	1.4	20	57	2004	417	N
*C. albicans*	Ward	7	1.1	12	9	2003	667	N
*C. albicans*	Ward	2	0.0	0	2	2005	588	N
*C. albicans*	Multiward	14	2.6	51	36	2005	10,000	N
*C. krusei*	Ward	2	0.3	7	11	2002	10,000	N
*C. lusitaniae*	Hospital-wide	2	0.0	0	1	2002	370	N
*T. (Candida) glabrata*	Ward	4	0.4	24	1	2003	1,250	N

a Number of days from the first culture associated with the cluster and the date of the first alert.

b Number of days between the first and the last alert for a cluster.

c Reflects the frequency (d) in which such as cluster is expected to occur by chance alone. Only clusters meeting a threshold recurrence interval of ≥365 d are provided.

**d–e:** Indicates same cluster identified by more than one signal type.

N, no; Y, yes.

Half of the detected clusters were gram-negative organisms not routinely tracked by Infection Control. In addition, 71% of clusters were identified by spatial characteristics other than traditional ward-based location, including groups of wards and services that shared patients and antimicrobial susceptibility patterns. The most common alerts (41%) were triggered by antibiotic resistance profiles. VRE clusters (*n* = 4) comprised 57% of enterococcal clusters and none were identified by ward-level spatial analyses (all were geographically dispersed, but shared antibiotic susceptibility profile). MRSA clusters comprised 58% of *S. aureus* alerts, and only three of seven clusters were based upon ward analyses (Table 3).

### Comparison with Clusters Previously Detected by Routine Infection Control Methods

Clusters identified using WHONET-SaTScan were compared to clusters previously identified through routine infection control surveillance. Other than pathogens identified by rule-based criteria that were evaluated separately (see below), all clusters previously identified and confirmed by the BWH Infection Control Department were also identified by WHONET-SaTScan. During the study period, the BWH Infection Control department identified two major clusters involving multidrug-resistant *Acinetobacter* (2004) and *Burkholderia cepacia* (2005), both of which were confirmed as clonal by pulse-field gel electrophoresis (PFGE). Both clusters were identified by WHONET-SaTScan within 3 and 6 d, respectively, of the initial isolate collection date.

**Figure 1:**
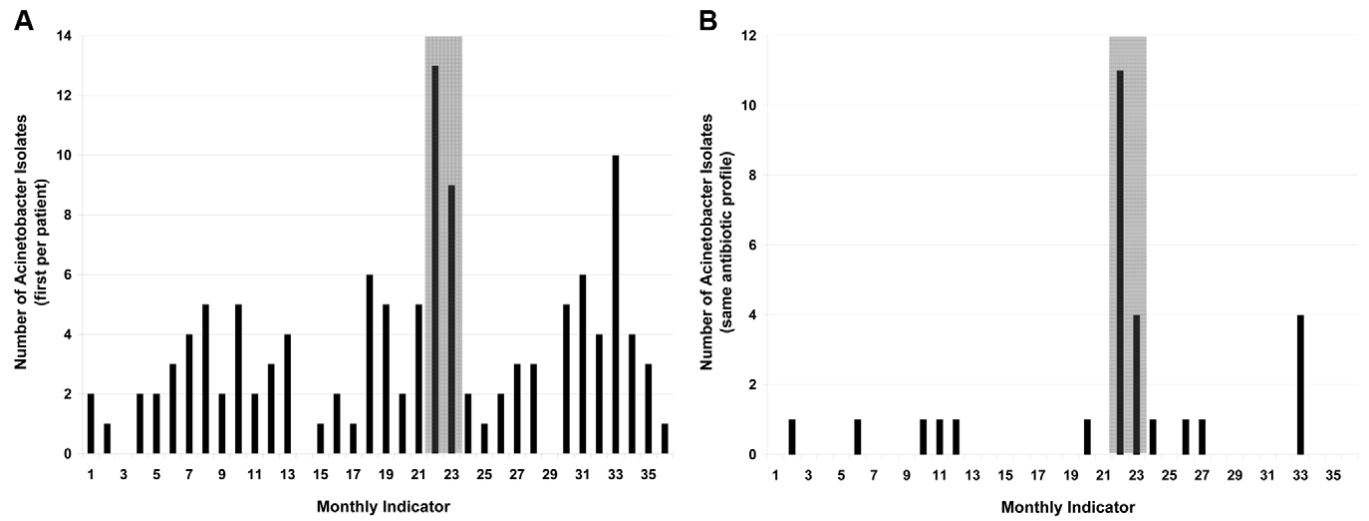
Display of monthly nosocomial *A. baumanii* isolates. (A) Hospital-wide. (B) Restricted to isolates with an identical antibiotic susceptibility profile. Shaded area in gray indicates time period of cluster detection by WHONET-SaTScan.

The clonal cluster of multidrug-resistant *Acinetobacter baumanii* involved patients in several intensive care units. WHONET-SaTScan identified this cluster through hospital-wide clustering of *A. baumanii* isolates (Figure 1A) as well as through clustering of a specific antimicrobial susceptibility pattern (Figure 1B).

In contrast, the BWH Infection Control department only identified three of the 59 clusters deemed to be statistically significant events on the basis of WHONET-SaTScan (Table 3, last column). Two coincided with the two clusters described above, and one involved MRSA.

### Comparison with Clusters Based on Numerical Thresholds

We compared the results of the WHONET-SaTScan statistical clusters to the rule-based criteria (i.e., ≥3 new nosocomial cases on a single ward within 2 wk) that were used by the Infection Control Department for MRSA and VRE during the study period. Many more MRSA alerts were triggered by the rule-based criteria (*n* = 73) versus WHONET-SaTScan statistical thresholds (*n* = 7), and only one of them was in common. Of interest, the one in common was a fairly large cluster of eight nosocomial isolates in an intensive care unit. No isolates were sent for genetic typing. Over half of the WHONET-SaTScan alerts were triggered by spatial analyses other than a single ward. Four alerts had a recurrence interval >1,000, and two reached the highest possible recurrence interval allowed by our parameter settings (10,000).

Similarly, many more VRE alerts were triggered by rule-based criteria (*n* = 87) versus WHONET-SaTScan statistical thresholds (*n* = 4). None of the alerts overlapped when methods were compared. Details of MRSA and VRE clusters detected by both methods are provided in Table 4. No additional overlap in MRSA or VRE clusters was identified when the recurrence interval was lowered to 200.

**Table 4:** Characteristics of MRSA and VRE clusters detected by routine infection control surveillance compared to WHONET-SaTScan.

Cluster Time Period	Infection Control Detection				WHONET-SaTScan Detection				Dual Detection
	*n* Clusters	Cases (Mean)	Duration (Mean Days)	Cluster Type^a^	*n* Clusters	Cases (Mean)	Duration (Mean Days)	Cluster Type	*n* Clusters
**MRSA**									
2002	14	10.8	96.5	Ward	1	14	67.0	Antibiotic profile	0
2003	11	11.1	100.3	Ward	0	—	—	—	0
2004	18	6.9	65.3	Ward	1	8	54.0	Ward	1
2005	18	5.9	52.4	Ward	3	3.7	8.3	Ward, ward/service, antibiotic profile	0
2006	12	4.9	48.0	Ward	2	4	6.0	Service, antibiotic profile	0
5-y total	73	—	—	—	7	—	—	—	1
**Annual mean**	14.6	7.9	72.5	—	1.4	5.9	27.1	—	0.2
**Annual median**	14	6.9	65.3	—	1.0	4.0	8.3	—	0
**VRE**									
2002	15	7.6	71.2	Ward	2	5.5	43.0	Antibiotic profile	0
2003	12	6.4	62.8	Ward	1	4.0	18.0	Antibiotic profile	0
2004	20	8.2	74.1	Ward	1	2.0	17.0	Antibiotic profile	0
2005	18	7.2	69.1	Ward	0	—	—	—	0
2006	22	6.0	58.3	Ward	0	—	—	—	0
5-y total	87	—	—	—	4	—	—	—	0
**Annual mean**	17.4	7.1	67.1	—	0.8	2.3	15.6	—	0
**Annual median**	18	7.2	69.1	—	1	2	17	—	0

a Infection Control identification of clusters was limited to wards only.

Only two rule-based clusters were deemed sufficiently large or persistent by the BWH Infection Control Department to warrant sending isolates for typing. Both involved MRSA. One occurred in the 2001 dataset that was used for parameterization (thus, not provided in Table 5). This cluster was rapidly detected by WHONET-SaTScan. The other cluster was an intensive care unit cluster in 2004 that was not detected by WHONET-SaTScan. This cluster involved nine nosocomial cases, but genetic typing revealed six different strain types, and the Infection Control Department ultimately ruled that this was not an outbreak.

**Table 5:** Correlation of two hospital epidemiologists independently assessing WHONET-SaTScan clusters.

	Ignore	Watch	Investigate	Actively Intervene	Total
**Ignore**	25	11	1	0	37 (63%)
**Watch**	2	5	1	2	10 (17%)
**Investigate**	0	0	0	0	0 (0%)
**Actively Intervene**	1	0	1	10	12 (20%)
**Total**	28 (47%)	16 (27%)	3 (5%)	12 (20%)	59 (100%)

### Assessing Utility and Response to Daily Alerts

**Figure 2:**
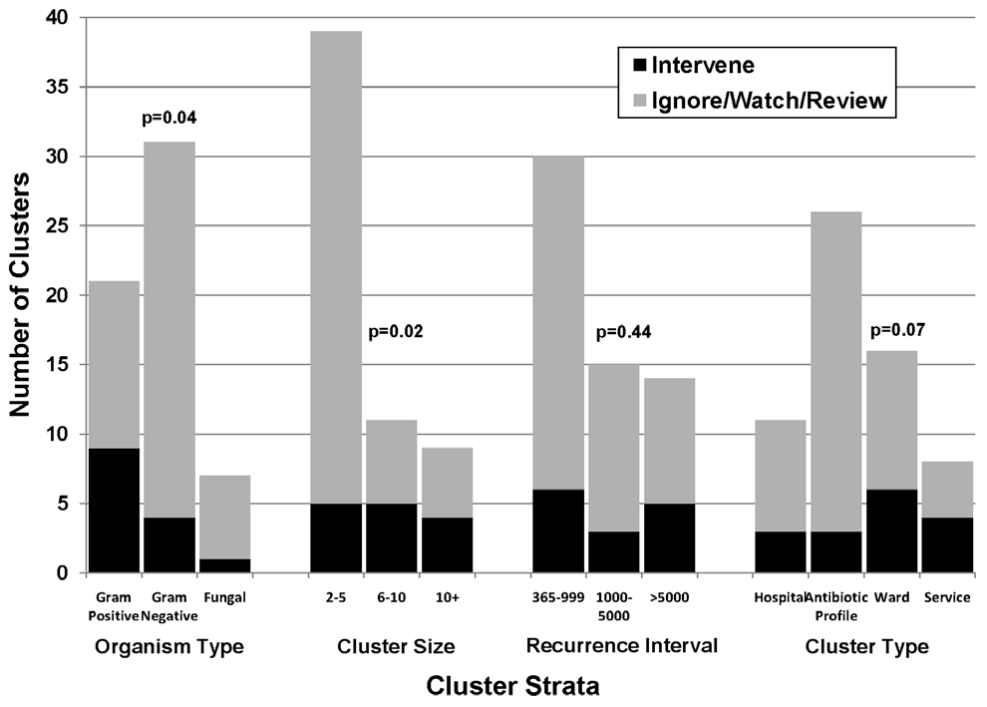
Graph showing survey-based Infection Control response by type of WHONET-SaTScan cluster. Significant differences among organism type and cluster size were noted when assessing the likelihood of triggering an intervention (Fisher exact tests). A trend toward a significant difference was found among cluster types. Among organism type, the likelihood of a cluster triggering an intervention was: gram-positive (43%), gram-negative (13%), fungal (14%). Among cluster size, the likelihood of a cluster triggering an intervention was: 2–5 (13%), 6–10 (45%), 10+ (44%). Among recurrence interval, the likelihood of a cluster triggering an intervention was: 365–999 (20%), 1,000–5,000 (20%), >5,000 (36%). Among cluster type, the likelihood of a cluster was: hospital (27%), antibiotic profile (12%), ward (38%), and service (50%).

The hospital epidemiologists classified 95% of the 59 cluster alerts as useful information. Sixteen (27%) of the clusters were classified as warranting either investigation or active intervention by at least one epidemiologist and 11(19%) by both (kappa = 0.76, confidence interval 0.5–0.8). The remaining 43 (73%) clusters were classified as warranting either no action or watchful waiting by both epidemiologists (Table 5). There were four clusters where the two epidemiologists disagreed about initiating active intervention. The reason for the discrepancies were due to a low number of events leading one epidemiologist to await further cases before acting while the other initiated intervention because of the significance of the pathogens (*aspergillus*, *pseudomonas*) or the source of the isolates (bacteremias). Certain cluster characteristics were associated with the likelihood of initiating active intervention (Figure 2).

## Discussion

The automated WHONET-SaTScan cluster detection tool rapidly detected epidemiologically confirmed hospital outbreaks in a large academic medical center and demonstrated that the common use of rule-based criteria (i.e., ≥3 new nosocomial cases on a single ward within 2 wk) for identifying clusters of MDROs often led to the identification of events likely to occur because of normal random fluctuations. Using a statistical method for cluster detection can focus hospital epidemiology efforts and conserve resources for events likely to represent actual outbreaks.

Current methods for cluster detection in hospitals are labor-intensive, narrow in focus, and subject to both over- and under-ascertainment of clusters. We linked two publicly available software systems to screen microbiology data for statistically significant clusters among all pathogens, across all wards and services.

In a single center study, we introduced the WHONET-SaTScan cluster detection tool and showed that it outperforms current infection control surveillance systems in several ways. First, it is more comprehensive. It is able to evaluate all pathogens with the potential to produce hospital-associated clusters. Current infection control surveillance is heavily focused on a small number of highly antibiotic-resistant bacteria, most of which are gram-positive pathogens. We found that two-thirds of identified clusters were due to gram-negative or fungal pathogens not under routine surveillance.

Second, the automated nature of WHONET-SaTScan makes it labor-sparing compared to usual surveillance, which identifies clusters from daily microbiologic feeds using the trained human eye. This software can be run daily within seconds and can provide a prospective tool for real-time cluster detection. Furthermore, the use of routinely available microbiologic data makes it adaptable by all hospitals using conventional microbiologic data systems. More importantly, it has the potential to spare the labor of unnecessary investigation of perceived clusters that are merely chance aggregations. These perceived clusters often result in substantial intervention costs and efforts on behalf of infection control and involved hospital wards.

Third, WHONET-SaTScan provides a statistical basis for cluster identification, thus improving the likelihood that the clusters represent health care–associated transmission events. When compared to conventional surveillance that uses numerical thresholds (rule-based criteria such as three cases in 2 wk in a single ward), we found that there was no significant statistical basis for nearly all of the clusters identified by routine infection control surveillance. This finding is not surprising given the rise in prevalence of MRSA and VRE—pathogens to which these rules are applied. In the example of MRSA, not only did rule-based criteria identify a large number of clusters (∼14/y) that may not have been real, but it failed to identify the once-a-year occurrence of a highly statistically significant cluster. Findings were even more striking for VRE. Although we recognize that statistical significance should not be the sole driver of cluster detection and response, the large discrepancy between statistically identified clusters and those found by infection control rule-based criteria suggests that statistical alerts (and lack of alerts) may provide a critical piece of information to guide action.

These results suggest that much of current infection control surveillance for nosocomial clusters may be ineffective, failing to find true clusters that may indicate unusual nosocomial transmission and identifying numerous events that likely represent random variation from a baseline rate as clusters that warrant resource-intensive investigation and response. The reduction in the number of MRSA and VRE clusters more than offset the increased number of clusters that resulted from identifying clusters caused by all pathogens. If this is a typical result, then statistically based surveillance could provide a major redirection of scarce infection control efforts. Notably, WHONET-SaTScan was able to identify the major pathogen clusters known to infection control that had clear epidemiologic links and evidence of genetic clonality.

Finally, the predictive value of alerts based on this scanning technique was acceptably high. Nearly all reported clusters were deemed of interest by the two hospital epidemiologists, and >25% generated sufficient concern to initiate an active investigation or full-scale intervention.

There are several limitations to this evaluation. First, it is a single center study providing subjective evaluation by two hospital epidemiologists, both of whom have been affected by prior experience at that hospital. Additional assessment in other centers is needed for validation.

Specifically, prospective validation is needed to evaluate whether statistical clusters are sufficiently important to warrant action, and whether ignoring rule-based clusters leads to no harm. In this study, we placed a subjective value on the WHONET-SaTScan clusters and assumed that all infection control clusters were deemed of high value. It was not possible to similarly assess the infection control clusters since action was taken once rule-based criteria were met. In addition, the recurrence interval was part of the assessment of WHONET-SaTScan clusters and this was not available for infection control clusters. The discrepancy between the WHONET-SaTScan results and the rule-based clusters can only be known in a prospective fashion when knowledge of statistical alerts can be integrated with clinical judgment to determine if action will be taken, and if a large cluster ensues because of inaction. If the value of statistical alerts continues to be demonstrated, then WHONET-SaTScan may provide a valuable tool for standardizing outbreak detection and evaluating the impact of various interventions to reduce nosocomial transmission.

Beyond further validation, this work requires replication and assessment of generalizability in other hospitals. Nevertheless, because it bases cluster determination on expected numbers of cases from recent history, it is adaptable to the varying conditions across institutions and the changing rates of pathogen colonization and infection. In this analysis, we identified clusters by comparing cluster case counts to the “spatial” and temporal locations of all other cases occurring during a 365-d period. Although this identification allows the analyses to be robust to secular trends in the prevalence of pathogens arising from different wards and services, other baseline periods could have been selected.

Secondly, clustering does not prove that there is an important biologic connection between cases. No matter what recurrence interval is selected, some clusters with a lower recurrence interval will reflect hospital transmission and some that exceed the value will be chance events. We do not have a precise estimate of this frequency because we performed a large number of scans across all pathogens and spatial dimensions. Further evaluation is needed to ensure that the threshold does not yield an unacceptable number of signals that are deemed of no interest. In this instance, the average of 12 alerts per year was far fewer than the number of clusters currently being identified by the Infection Control department. Notably, in this study, lowering the statistical threshold did not increase the overlap between WHONET-SaTScan clusters and those found by infection control.

In conclusion, we demonstrate the usefulness of automated cluster detection that uses readily available microbiology data to identify clusters of clinically relevant nosocomial pathogens. This approach to cluster detection has the potential to be more comprehensive than current surveillance systems and save substantial amounts of infection control resources [14]. Most importantly and provocatively, these findings suggest that many of the events that trigger outbreak control protocols probably represent random variation rather than true outbreaks. Additionally, current infection control methods fail to identify a majority of events that are statistically unusual and may represent opportunities for intervention. This statistically based cluster detection tool could be readily implemented to improve and streamline the daily practice of infection control professionals.

## Abbreviations

BWH: Brigham & Women's Hospital
MDRO: multidrug-resistant organism
MRSA: methicillin-resistant *Staphylococcus aureus*
VRE: vancomycin-resistant enterococci
WHO: World Health Organization
